# EWAS Data Hub: a resource of DNA methylation array data and metadata

**DOI:** 10.1093/nar/gkz840

**Published:** 2019-10-04

**Authors:** Zhuang Xiong, Mengwei Li, Fei Yang, Yingke Ma, Jian Sang, Rujiao Li, Zhaohua Li, Zhang Zhang, Yiming Bao

**Affiliations:** 1 National Genomics Data Center, Beijing 100101, China; 2 BIG Data Center, Beijing Institute of Genomics, Chinese Academy of Sciences, Beijing 100101, China; 3 CAS Key Laboratory of Genome Sciences and Information, Beijing Institute of Genomics, Chinese Academy of Sciences, Beijing 100101, China; 4 College of Life Sciences, University of Chinese Academy of Sciences, Beijing 100049, China; 5 School of Future Technology, University of Chinese Academy of Sciences, Beijing 100049, China

## Abstract

Epigenome-Wide Association Study (EWAS) has become an effective strategy to explore epigenetic basis of complex traits. Over the past decade, a large amount of epigenetic data, especially those sourced from DNA methylation array, has been accumulated as the result of numerous EWAS projects. We present EWAS Data Hub (https://bigd.big.ac.cn/ewas/datahub), a resource for collecting and normalizing DNA methylation array data as well as archiving associated metadata. The current release of EWAS Data Hub integrates a comprehensive collection of DNA methylation array data from 75 344 samples and employs an effective normalization method to remove batch effects among different datasets. Accordingly, taking advantages of both massive high-quality DNA methylation data and standardized metadata, EWAS Data Hub provides reference DNA methylation profiles under different contexts, involving 81 tissues/cell types (that contain 25 brain parts and 25 blood cell types), six ancestry categories, and 67 diseases (including 39 cancers). In summary, EWAS Data Hub bears great promise to aid the retrieval and discovery of methylation-based biomarkers for phenotype characterization, clinical treatment and health care.

## INTRODUCTION

Epigenome-Wide Association Study (EWAS) has become an effective strategy to explore epigenetic basis of complex traits, such as aging (1–4), body mass index (BMI) (5,6), smoking (7,8) and diseases (9,10), accordingly leading to massive amounts of epigenetic data. Among different types of epigenetic data, DNA methylation is the most abundant and widely characterized one, primarily owing to the rapid advancement in DNA methylation profiling technologies, especially Infinium HumanMethylation450 (450K) and MethylationEPIC (850K) arrays (11,12). Therefore, comprehensive integration of DNA methylation array data and metadata is of fundamental significance to systematically characterize and investigate methylation states across different experimental conditions and explore epigenetic mechanisms associated with diverse traits.

Over the past several years, several databases have been developed to host DNA methylation array data (13–19), primarily including Gene Expression Omnibus (GEO) (20), ArrayExpress (21), The Cancer Genome Atlas (TCGA) (22), Encyclopedia of DNA Elements (ENCODE) (23), Firehose and The cBio Cancer Genomics Portal (cBioPortal) (24). Although they made valuable efforts to help users conduct methylation studies, these databases have three significant drawbacks. First, they lack an effective and unified normalization method to remove batch effects among different datasets, which may exert severe negative influences on downstream analysis (25,26). Second, different databases use different metadata standards, making it challenging to integrate methylation data across diverse conditions and samples. Third, as a result, none of them provides standardized and normalized DNA methylation profiles across different tissues, sexes, ancestry categories and diseases. In short, these databases are designed mainly for archiving raw data, without value-added curation for data normalization and metadata standardization.

To address these drawbacks, we develop EWAS Data Hub (https://bigd.big.ac.cn/ewas/datahub), for collecting and normalizing DNA methylation array data as well as archiving associated metadata. More than just rehosting datasets as they appear in public databases, a pipeline optimized for data normalization and metadata curation is employed to remove batch effects and standardize metadata across different datasets. Thus, EWAS Data Hub not only provides normalized methylation data and standardized metadata but also integrates a comprehensive collection of high-quality methylation profiles across different contexts.

## IMPLEMENTATION

EWAS Data Hub is implemented using Spring Boot (http://spring.io), a prevailing and easy-to-configure Model-View-Controller (MVC) framework, deployed in a Centos Linux 7.4 environment. Thymeleaf (https://www.thymeleaf.org), which is integrated with the Spring Framework, is used to render the HTML interface. In the back-end part, metadata and reference data are stored in MySQL (https://www.mysql.com). Front-end interfaces are built using Bootstrap (https://getbootstrap.com) with jQuery (https://jquery.com) to provide responsive and user-friendly web pages. The documentation is generated by docsify (https://docsify.js.org). HighCharts (https://www.highcharts.com) and plotly (https://plot.ly/) are used to provide interactive charting and data visualization.

## DATA CURATION AND DATABASE CONTENTS

**Figure 1.**
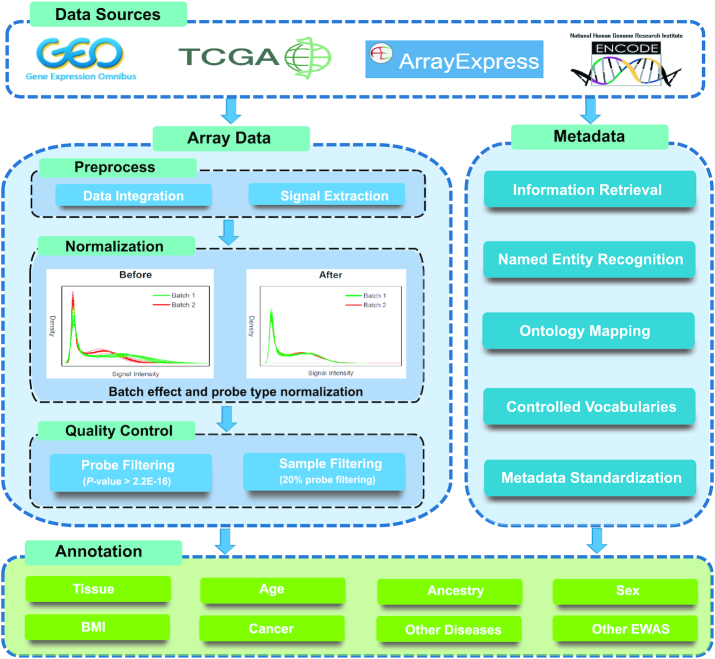
Schematic overview of data processing workflow.

We download all available datasets generated by Infinium HumanMethylation450 or MethylationEPIC arrays from GEO, TCGA, ArrayExpress and ENCODE. If raw data are available, signal intensities are extracted using the Minfi package in Bioconductor (27). A series of curation processes are applied to remove batch effects and improve the quality of data (Figure 1). First, we normalize signal intensities of type I probes between arrays using an in-house reference-based method (for details see the Documentation in the database website). Second, Beta-Mixture Quantile Normalization (BMIQ) is employed to correct the bias associated with technical differences between Type I and Type II array designs (28). Previous studies have shown that setting a stringent detection *P*-value threshold (10^−16^) is able to significantly reduce the proportion of outlying values (due to a low signal-to-noise ratio of fluorescence intensities) and accordingly achieve improved calling (29,30). Therefore, we perform rigorous quality control to filter out probes with high detection *P*-values (by default, the threshold is set at 2.2 × 10^−16^, which is the smallest number that can be stored by the floating system in R program) and remove samples with more than 20% of the probes with high detection *P*-values (31). To standardize the metadata, we develop a curation model that summarizes 178 fields, including four common fields (sample ID, project ID, tissue/cell and sample type which refers to health status of the sample) and 174 other fields (sex, age, disease, etc.) (see the online Documentation for details). We standardize the values of all fields, if applicable, by setting up controlled vocabularies or mapping them to experimental factor ontology (EFO) that combines parts of several biological ontologies, such as Disease Ontology (DO) and Gene Ontology (GO).

**Figure 2.**
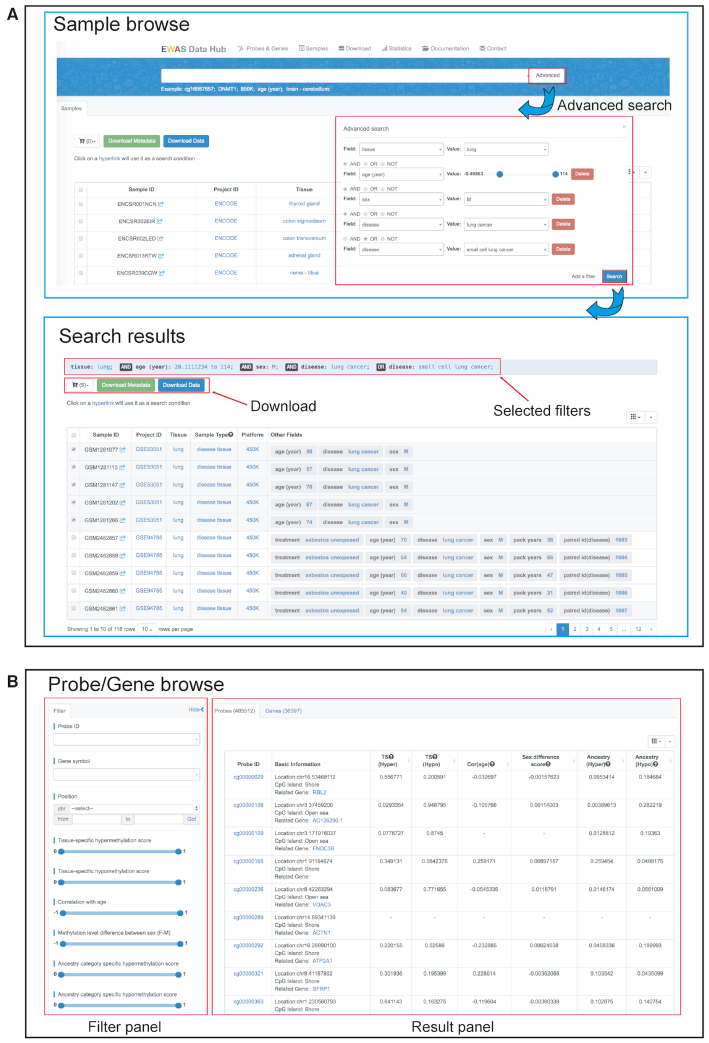
Screenshots of the ‘Browse’ pages. (**A**) An example of advanced search and its results, (**B**) the ‘Browse’ page of probe/gene.

Based on the standardized curation process, EWAS Data Hub obtains a comprehensive collection of DNA methylation data as well as associated metadata from 75 344 samples (Figure 2), including 470 tissues/cell types, 306 diseases and other conditions. In order to find the sample(s) with specific characteristics, a set of advanced filters, such as tissue, age, sex and platform, are provided to facilitate users to query and narrow down the searched results (Figure 2A). After retrieval, users can download data and metadata of retrieved samples. Importantly, EWAS Data Hub integrates a curated collection of 485 512 probes in association with 36 397 genes (Figure 2B). For each probe/gene, it provides a series of relevant estimates, including tissue-specificity, age correlation, sex difference and ancestry-specificity. In addition, EWAS Data Hub is equipped with multiple filters, allowing users to easily find probes and genes of interest. Specifically, these filters include tissue-specificity score estimated across all collected tissues, correlation coefficient with age, methylation level difference between sex, and ancestry-specificity score. Taken together, EWAS Data Hub features a complete collection of curated probes/genes as well as standardized sample metadata.

**Figure 3.**
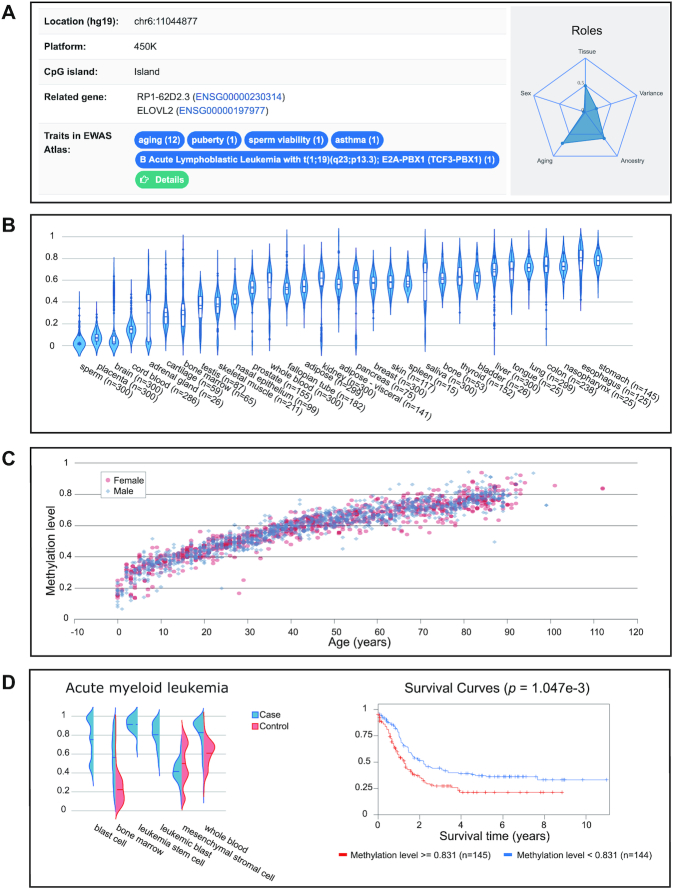
Reference data of probe ‘cg16867657’. (**A**) The ‘Basic’ panel, (**B**) the ‘Tissue’ panel, (C) the ‘Age’ panel and (**D**) the ‘Cancer’ panel.

For each probe/gene, EWAS Data Hub provides reference DNA methylation profiles cross different contexts, making it possible to systematically characterize and investigate the landscape of methylation states across a wide range of experimental conditions. To facilitate data presentation, taking the probe ‘cg16867657’ (https://bigd.big.ac.cn/ewas/datahub/probe/cg16867657) as an example, all these data are organized into different panels in terms of basic, tissue, sex, age, ancestry category, BMI, cancer, disease and public EWAS, respectively (Figure 3).

The ‘Basic’ panel not only provides fundamental information such as genomic location, position relative to CpG island, associated phenotypic traits in EWAS Atlas (32), but also summarizes the correlation of DNA methylation level with tissue, sex, age and ancestry (Figure 3A). Besides, a genome browser is presented to visualize related data in an interactive manner. The ‘Tissue’ panel contains DNA methylation profiles across 31 tissues, 25 brain parts and 25 blood cell types (Figure 3B). For each probe, its methylation profiles across different tissues/brain parts/blood cell types are depicted in a violin plot, which can greatly help users explore the methylation pattern in various conditions. The ‘Sex’ panel provides DNA methylation profiles across different sexes, which would be helpful to investigate the heterogeneity of DNA methylation in male and female. The ‘Age’ and ‘BMI’ panels provide the distribution of DNA methylation by chronological age and BMI, respectively (Figure 3C). Following a previous study (28), six ancestry categories are adopted in our study. Therefore, the ‘Ancestry’ panel contains six categories by grouping all datasets into different ancestries. The ‘Cancer’ and ‘Other disease’ panels provide DNA methylation profiles across 39 cancers and 28 diseases in both case and control samples. For cancers, Kaplan-Meier survival analyses of overall survival of patients according to the DNA methylation status are conducted (Figure 3D). Moreover, the relationships between DNA methylation and expression of proximal genes are presented in a scatter plot. The Public EWAS panel provides detailed information of public EWAS associations. In partnership with EWAS Atlas (32), EWAS Data Hub automatically retrieves related traits for each probe/gene and provides users with convenient links to EWAS Atlas. Moreover, all datasets collected in this study are publicly available at https://bigd.big.ac.cn/ewas/datahub/download.

## DISCUSSION AND FUTURE DEVELOPMENTS

Considering the significance of DNA methylation as one of the most promising cancer diagnostic and therapeutic targets and also a key link between environmental factors and phenotypes (33–38), EWAS Data Hub provides great opportunities to dissect epigenetic mechanisms underlying complex biological traits by integrating and normalizing large amounts of DNA methylation array data as well as curating and standardizing the corresponding metadata. With the ever-growing volume of DNA methylation data and the rapid development of methylation profiling technology, EWAS Data Hub will be updated regularly to integrate more DNA methylation array data, especially those from 850K. Accordingly, the reference DNA methylation profiles will be updated and expanded to include more phenotypic traits, making it possible to conduct a meta-analysis for probes and genes from multiple studies of the same trait. In addition, considering that DNA methylation in combination with gene expression pattern has been frequently used to explore the molecular mechanisms between epigenetics and phenotype (39,40), the relationships between DNA methylation and expression of proximal genes in more phenotypes will be added to EWAS Data Hub. Moreover, online tools to visualize and analyze DNA methylation array data will be developed.
